# Force Map-Enhanced Segmentation of a Lightweight Model for the Early Detection of Cervical Cancer

**DOI:** 10.3390/diagnostics15050513

**Published:** 2025-02-20

**Authors:** Sabina Umirzakova, Shakhnoza Muksimova, Jushkin Baltayev, Young Im Cho

**Affiliations:** 1 Department of Computer Engineering, Gachon University, Sujeong-gu, Seongnam-si 461-701, Gyeonggi-do, Republic of Korea; sabinatuit@gachon.ac.kr; 2 Department of Information Systems and Technologies, Tashkent State University of Economic, Tashkent 100066, Uzbekistan; j_baltayev@tsue.uz

**Keywords:** cervical cancer segmentation, instance segmentation, lightweight model, medical imaging, multi-scale feature aggregation, deep learning

## Abstract

**Background/Objectives:** Accurate and efficient segmentation of cervical cells is crucial for the early detection of cervical cancer, enabling timely intervention and treatment. Existing segmentation models face challenges with complex cellular arrangements, such as overlapping cells and indistinct boundaries, and are often computationally intensive, which limits their deployment in resource-constrained settings. **Methods:** In this study, we introduce a lightweight and efficient segmentation model specifically designed for cervical cell analysis. The model employs a MobileNetV2 architecture for feature extraction, ensuring a minimal parameter count conducive to real-time processing. To enhance boundary delineation, we propose a novel force map approach that drives pixel adjustments inward toward the centers of cells, thus improving cell separation in densely packed areas. Additionally, we integrate extreme point supervision to refine segmentation outcomes using minimal boundary annotations, rather than full pixel-wise labels. **Results:** Our model was rigorously trained and evaluated on a comprehensive dataset of cervical cell images. It achieved a Dice Coefficient of 0.87 and a Boundary F1 Score of 0.84, performances that are comparable to those of advanced models but with considerably lower inference times. The optimized model operates at approximately 50 frames per second on standard low-power hardware. **Conclusions:** By effectively balancing segmentation accuracy with computational efficiency, our model addresses critical barriers to the widespread adoption of automated cervical cell segmentation tools. Its ability to perform in real time on low-cost devices makes it an ideal candidate for clinical applications and deployment in low-resource environments. This advancement holds significant potential for enhancing access to cervical cancer screening and diagnostics worldwide, thereby supporting broader healthcare initiatives.

## 1. Introduction

Cervical cancer remains one of the leading causes of cancer-related mortality among women worldwide, particularly in low- and middle-income countries where access to preventive care and early diagnostic methods is limited. The early detection of precancerous and cancerous cells in cervical cytology images, such as those obtained from Pap smears, is crucial to preventing cervical cancer progression [1,2]. The accurate segmentation of cervical cells in these images is essential for identifying abnormal cells and assessing morphological changes that may indicate early-stage cancer [3]. However, manual analysis by pathologists is time-consuming and subjective, leading to variability in diagnoses [4]. This variability underscores the need for automated, reliable, and efficient segmentation methods to facilitate early diagnosis and assist pathologists in making informed decisions.

Automated cervical cell segmentation presents several technical challenges. Cervical cell images often contain multiple cells with varying morphology, size, and orientation [5]. Cells may be tightly packed, partially overlapping, or even occluding each other, complicating the distinction of individual cells. Furthermore, the boundaries of cells are sometimes faint or blurred due to staining variations and differences in image quality, making it difficult for traditional segmentation methods to accurately capture these boundaries. The presence of artifacts, noise, and variations in staining protocols adds another layer of complexity, requiring robust algorithms that can generalize well across diverse imaging conditions.

Existing deep learning methods, particularly convolutional neural networks (CNNs), have shown promising results in medical image segmentation [6]. However, many of these models are computationally expensive, relying on complex architectures with large numbers of parameters [7]. In resource-limited environments, such as mobile or embedded systems, these models are impractical due to their high computational and memory requirements.

To address this challenge, there is a need for lightweight segmentation models that can achieve high accuracy while operating efficiently on low-power devices, enabling broader accessibility and use in clinical settings. Given the constraints of real-world deployment, especially in low-resource healthcare settings, an effective cervical cell segmentation model should balance accuracy with efficiency. Lightweight models, which use fewer parameters and require less computational power, are crucial for applications in mobile health technologies or environments with limited access to high-end computing resources. By reducing the computational requirements without sacrificing performance, such models can make automated cervical cancer screening and diagnostics more accessible, especially in remote or underserved areas. Additionally, lightweight models facilitate real-time processing, which is valuable in clinical workflows where rapid decision making is often necessary.

In this study, we propose a lightweight and efficient segmentation model for cervical cell analysis that aims to achieve state-of-the-art (SOTA) performance with minimal computational overhead. Our model incorporates the following key innovations:

- We leverage the MobileNetV2 architecture as the model backbone due to its efficient design, which utilizes depthwise separable convolutions and inverted residuals. This structure significantly reduces the model’s parameter count and computational requirements while preserving its capability to extract essential features from high-resolution cervical cell images.
- To capture the diverse range of morphological features in cervical cell images, our model employs multi-scale feature aggregation. By combining feature maps from different levels of the model, we enhance its ability to identify both fine-grained cell details and broader structural patterns, allowing for accurate segmentation even in images with complex cell arrangements.
- To address the challenges posed by overlapping and densely packed cells, we introduce a force map and an extreme point-based boundary refinement mechanism. The force map directs pixels within each cell toward the cell’s center, enhancing boundary separation in overlapping regions. Additionally, the use of extreme points (the topmost, bottom-most, leftmost, and rightmost points of each cell) provides spatial anchors that guide the model to accurately delineate boundaries, even in challenging configurations.
- Our model is trained with weak supervision using minimal annotations, leveraging extreme points instead of full pixel-wise labels. This approach reduces the need for costly manual annotation while providing sufficient structural guidance for accurate segmentation, making the model more feasible for large-scale deployment.
- By designing the model to be lightweight, we achieve fast inference speeds, enabling real-time segmentation on low-power devices such as mobile or embedded platforms. This efficiency is especially beneficial in clinical and point-of-care settings where rapid analysis can enhance workflow and decision making.

Our work addresses the need for an accurate, efficient, and accessible cervical cell segmentation model by combining a lightweight architecture with advanced boundary refinement techniques. Our model strikes a balance between segmentation accuracy and computational efficiency, making it suitable for deployment in diverse clinical environments, including those with limited computational resources. Through extensive evaluation, we demonstrate that our model not only achieves competitive performance with SOTA methods but is also optimized for real-time applications in cervical cancer screening. To validate the robustness of our approach, we utilized the publicly available SipakMed Pap Smear dataset, which consists of 4049 high-resolution cervical cell images derived from 966 complete slide images. This dataset represents a diverse range of cytomorphological structures, including both healthy and abnormal cells. It encompasses five distinct categories—Dyskeratotic, Koilocytotic, Parabasal, Superficial–Intermediate, and Metaplastic cells—ensuring that our model is exposed to a wide variety of cellular features. The dataset’s complexity is further heightened by the presence of densely packed cells, overlapping cell regions, and faint or blurred boundaries, which mimic real-world diagnostic challenges. Additionally, the dataset’s annotations are limited to extreme points for each cell, reflecting the practical constraints of obtaining full pixel-wise annotations. These factors collectively underscore the need for segmentation methods that are not only accurate but also efficient and generalizable across diverse and complex cell images.

The remainder of this paper is organized as follows: Section 2 reviews related work in cervical cell segmentation and lightweight models in medical imaging. Section 3 provides a detailed description of the proposed model architecture, including the MobileNetV2 backbone, multi-scale feature aggregation, and force map-based boundary refinement. Section 4 outlines the dataset used, preprocessing steps, evaluation metrics, and implementation details. Section 5 presents and discusses our model’s quantitative and qualitative results, including comparisons with SOTA models. Finally, Section 6 concludes the paper by summarizing the contributions and suggesting directions for future research.

## 2. Literature Analysis

Automated cervical cell segmentation has attracted significant research interest due to its critical role in early cervical cancer detection [8]. Although various deep learning techniques have advanced medical image segmentation, achieving efficient, accurate, and robust segmentation in complex cell images remains a challenging task. In this section, we review related works in three main areas: (1) traditional methods and CNNs for cervical cell segmentation; (2) recent advancements in lightweight and efficient deep learning architectures; and (3) boundary refinement techniques used to improve segmentation accuracy in densely packed cell regions.

### 2.1. Traditional Methods and CNNs for Cervical Cell Segmentation

Early efforts in cervical cell segmentation relied on traditional image processing methods, such as thresholding, morphological operations, and watershed algorithms, to separate cell regions from the background. Techniques based on color, texture, and shape descriptors, combined with region-growing methods, were commonly used to identify and segment individual cells. However, these approaches typically lacked robustness in handling complex scenarios, such as overlapping cells, low-contrast boundaries, and staining variations, which are common in cervical cytology images. With the advent of deep learning, CNNs quickly became the preferred choice for medical image segmentation [9]. U-Net, a fully convolutional architecture, gained widespread popularity for its encoder-decoder structure and skip connections, which enable it to capture both local and global features [10]. Several variants of U-Net have been proposed to enhance segmentation accuracy for cell images. For example, approaches incorporating attention mechanisms, such as Attention U-Net [11,12], allow the network to focus more precisely on regions of interest (see Algorithm 1).
**Algorithm 1** Key Preprocessing operations1:    function PREPROCESS_CERVICAL_IMAGE(I) 2:           I_lab ← ConvertToLAB(I) 3:           clusters ← KMeansClustering(I_lab, k = 3) 4:           I_segmented ← ExtractForeground(clusters) 5:         I_texture ← ApplyGaborFilters(I_segmented) 6:         I_texture ← ApplyLBP(I_texture) 7:         I_cleaned ← MorphologicalOpen(I_texture, kernel = 3 × 3) 8:         I_cleaned ← MorphologicalClose(I_cleaned, kernel = 5 × 5) 9:         (top, bottom, left, right) ← ExtractExtremePoints(I_cleaned) 10:         return (I_cleaned, top, bottom, left, right) 11: end function

However, while these models offer significant improvements in accuracy, their large number of parameters and high computational requirements limit their applicability in resource-constrained environments. Specifically, in cervical cell segmentation, CNN-based methods such as DeepLab and Mask R-CNN have shown promise in identifying individual cell boundaries and handling complex morphologies [13,14]. These models leverage multi-scale feature extraction and often integrate additional modules for improved boundary detection. However, these architectures are typically computationally intensive, making them challenging to deploy in real-time applications or on devices with limited processing power. This limitation has motivated research into lightweight, efficient models capable of achieving high segmentation performance with fewer resources.

### 2.2. Lightweight and Efficient Deep Learning Models

The need for efficient and deployable models has driven research toward lightweight architectures, particularly for applications in mobile and embedded systems [15]. MobileNet [16], SqueezeNet [17], and ShuffleNet [18] are among the most popular architectures designed for efficiency. These models achieve high performance by using techniques such as depthwise separable convolutions, parameter reduction, and compressed network structures. MobileNetV2 [19], in particular, has gained popularity for its use of inverted residuals and linear bottlenecks, which allow it to retain accuracy while drastically reducing computational complexity. This architecture has shown success in various computer vision tasks and has been adapted for medical imaging applications. Recent studies have explored the application of MobileNet-based models for segmentation tasks, showing that these lightweight architectures can be effective in low-resource environments. For example, Mobile-UNet and Fast-SCNN incorporate the lightweight structure of MobileNet with additional layers to enhance segmentation accuracy while minimizing computational load [20]. These models have been applied to tasks such as skin lesion segmentation and retinal vessel segmentation, demonstrating that efficient models can achieve high accuracy in medical imaging. However, lightweight models in cervical cell segmentation remain underexplored. Most existing works focus on large, resource-intensive architectures, as they prioritize accuracy over efficiency. Our work addresses this gap by combining a MobileNetV2 backbone with multi-scale feature aggregation and boundary refinement techniques tailored to cervical cell images. This combination allows for an effective balance between accuracy and computational efficiency, making it suitable for deployment in real-time and resource-limited settings.

### 2.3. Boundary Refinement Techniques for Cell Segmentation

Boundary precision is essential for accurate segmentation in cervical cell images, particularly when dealing with overlapping or densely packed cells [21]. Traditional CNN architectures may struggle to delineate cell boundaries in these scenarios, leading to merged or incomplete segmentations. To improve boundary accuracy, several boundary refinement techniques have been proposed in recent years [22]. One common approach is to use contour-based loss functions, which penalize the network for errors along the boundaries of segmented objects. For example, Boundary IoU and Hausdorff distance-based losses are frequently used to enforce sharper boundaries in medical image segmentation. These losses encourage the model to focus more on boundary regions, reducing the overlap and merging issues that often occur in densely packed images. Another effective boundary refinement method is the use of attention mechanisms. For instance, Attention U-Net uses spatial attention to enhance feature maps along boundary regions, helping the model to better distinguish between adjacent cells [23]. This method improves the network’s sensitivity to cell edges, particularly in regions with low contrast or faint boundaries. Additionally, approaches like the Contour-Aware Network (CANet) integrate contour information directly into the network, helping to separate closely situated cell regions by explicitly modeling boundary features [24]. In cervical cell segmentation, where accurate boundary delineation is essential, these techniques are highly beneficial but are often computationally demanding. In our work, we introduce a novel force map and extreme point-based boundary refinement strategy that achieves precise boundary delineation with minimal computational cost. Inspired by physical force models, our force map guides pixels within each cell toward the center, enhancing separation between adjacent cells, while the extreme point annotations (top, bottom, left, and right) provide spatial anchors for accurate boundary refinement. This approach enables our model to achieve high boundary accuracy even in complex cell configurations, without relying on computationally expensive modules. While traditional CNN architectures and boundary refinement techniques have made significant strides in improving segmentation accuracy, their computational demands limit practical application, particularly in resource-constrained settings. Lightweight models such as MobileNet and efficient segmentation networks are emerging as viable alternatives, but their application in cervical cell segmentation is still limited. Our proposed model addresses these gaps by integrating a MobileNetV2 backbone with novel boundary refinement methods, specifically designed for cervical cell segmentation. This combination of efficiency and accuracy enables our model to perform well in real-time applications, making it a promising solution for cervical cancer screening and diagnostics in diverse clinical environments.

## 3. Methodology

**Figure 1:**
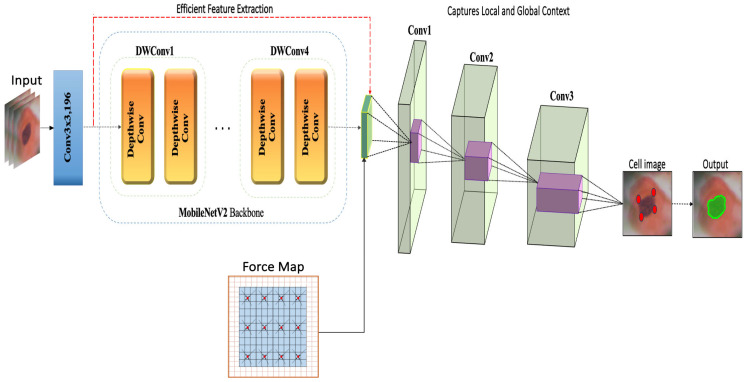
Improved contextual integration and feature extraction with MobileNetV2 for accurate cell segmentation.

Figure 1 illustrates a deep learning architecture employing the MobileNetV2 backbone for efficient feature extraction in images. This architecture utilizes depthwise separable convolutions along with additional convolutional layers to capture both local and global contexts within images. A “force map” is incorporated to enhance spatial analysis, which is crucial for the detection of detailed features, such as cells in medical images. The process yields a clearly delineated output that highlights the identified features. This configuration is ideally suited for applications requiring precise image analysis and rapid processing.

This paper presents a weakly supervised, multi-scale segmentation framework for cervical cancer cell classification, designed to address the unique challenges of cervical cell images, such as overlapping cells, varying morphologies, and indistinct boundaries. By integrating weak supervision, multi-scale feature extraction techniques, and force-directed boundary refinement, this framework achieves accurate segmentation with minimal annotation. The process begins with color- and texture-based clustering, which isolates cell regions from the background and generates initial pseudo-labels for coarse segmentation. To refine these labels without costly pixel-level annotation, we incorporate point-based supervision, marking only the cell’s extreme points (top, bottom, left, and right). These points help the model adjust boundaries, especially in regions where cells overlap. Next, our Vision Transformer (ViT)-based encoder aggregates multi-scale features, enabling the model to capture both small cellular details and broader morphological structures. This multi-scale approach enhances the segmentation accuracy across varied cell sizes and shapes, supporting the precise differentiation of abnormal cell regions. For further refinement, a force map assigns directional vectors to each cell’s pixels, guiding them toward the cell’s center. This force-directed method emphasizes boundaries between adjacent cells, enhancing precision with extreme points. This combination of directional forces and point-based guidance results in a refined segmentation that is particularly effective in crowded areas. This framework combines weak supervision and instance-specific refinement techniques to deliver high-quality segmentation with reduced annotation effort, providing an efficient foundation for cervical cancer classification and early detection.

### 3.1. Preprocessing

The preprocessing stage establishes a foundational segmentation by isolating cellular regions from the background in cervical images. We use color and texture-based clustering to distinguish cells from non-cellular areas, generating initial pseudo-labels that guide later stages of the segmentation process. Cells in cervical images often show distinct color and texture patterns. To capture these, we apply K-means clustering in a color space like HSV or LAB to highlight cellular regions, followed by texture analysis using Gabor filters or Local Binary Patterns (LBPs). This combination provides a robust initial segmentation, effectively separating potential cellular structures from background elements. With the foreground-background separation established, we create preliminary pseudo-labels to serve as coarse segmentation masks. These pseudo-labels undergo morphological refinement to clean up boundaries and remove artifacts. Connected component analysis further ensures that each cell cluster is treated as an individual instance, ready for the next segmentation phase. To streamline computations, we apply downsampling techniques on the foreground regions, reducing data density without compromising essential structure. This simplifies the initial input for the model, allowing it to focus efficiently on critical cellular details. Through this combination of clustering, pseudo-labeling, and sampling, the preprocessing stage reduces background noise and prepares the model for effective weakly supervised segmentation.

### 3.2. Weak Supervision and Semantic Point Annotations

In medical imaging, pixel-level annotations, for instance segmentation, are often costly and time-consuming to obtain, especially for large datasets. To address this, our framework employs a weakly supervised approach that leverages minimal yet highly informative annotations in the form of semantic point annotations, or “extreme points”, placed at strategic positions on each cell. Extreme points are particularly robust for model generalization as they capture essential morphological features of cells that remain invariant across variations in cell orientation, size, and density. This robustness ensures that the model learns boundary-critical features without being overly reliant on exhaustive pixel-wise annotations, which can introduce noise or overfitting. Furthermore, extreme points inherently focus the model learning capacity on key boundary regions, enabling effective segmentation even in challenging scenarios involving densely packed or overlapping cells. By relying on these key points alone, we can guide the model to accurately delineate cell boundaries, particularly in overlapping or densely packed regions, significantly reducing annotation costs without compromising accuracy. The weak supervision strategy uses four extreme points—specifically, the topmost, bottom-most, leftmost, and rightmost points around each cell. These points, representing the outer boundaries of each cell, provide crucial positional cues with minimal annotation effort. Unlike traditional pixel-level labeling, this sparse annotation method efficiently captures essential boundary information that directs the model in refining cell boundaries. Each extreme point serves as a positional anchor, helping the model infer the cell’s shape and orientation, even in cases of overlapping cells or those with ambiguous edges. Combined with the preliminary pseudo-labels generated during preprocessing, these annotations guide the model in distinguishing individual cells within complex arrangements. During training, extreme points are used as spatial anchors that refine the segmentation boundaries and extreme points are utilized as spatial anchors through a dedicated point distance loss function. This loss penalizes deviations between the predicted segmentation boundaries and the annotated extreme points by minimizing the Euclidean distance between them. The model learns to interpret these points as the cell’s outer limits, reinforcing boundary detection capabilities in dense clusters or partially obscured regions. To enforce accurate alignment of the model predictions with these boundary markers, we employ a point distance loss. The point distance loss penalizes deviations between the predicted boundary and the annotated extreme points, calculated using the Euclidean distance. For each cell *C_i_* with extreme points $\left\{p_{top},p_{bottom},p_{left},p_{right}\right\}$ and a predicted boundary $B_{i}$, the point distance loss $L_{pd}$ is defined as follows:

(1) $$L_{pd}=\sum_{p\in \{p_{top},p_{bottom},p_{left},p_{right}\}}\underset{q\in B_{i}}{\min}||p-{q||}^{2},$$

where *p* represents each extreme point, q represents points on the predicted boundary $B_{i}$, and ∥⋅∥ denotes the Euclidean distance. This loss function encourages the model to produce boundaries that closely match the extreme points, effectively guiding the segmentation process toward accurate boundary alignment. The extreme points not only serve as boundary markers but also enhance the model capacity to manage complex cell structures. By enforcing constraints around each cell instance, the annotations help refine boundaries, particularly in cases of cell overlap. When cells are densely packed, the extreme points act as constraints that prevent the model from merging adjacent cells, even if the actual boundaries are faint or indistinct.

For cells with irregular shapes or partial occlusion, the extreme points offer reliable boundary cues, allowing the model to extrapolate contours more accurately. This point-guided approach helps the model maintain the unique shape of each cell, providing precise segmentation even in challenging regions. Utilizing extreme points provides several benefits. First, it enables high segmentation accuracy with minimal annotation, making it feasible for larger datasets without exhaustive pixel-level labeling. Second, the extreme points provide spatially grounded cues for boundary delineation, crucial for resolving overlaps and maintaining instance separation. Lastly, by focusing on boundary-critical points, the model can better generalize across varying cell morphologies, capturing the diversity within cervical cell images. This weakly supervised, point-guided method effectively balances annotation efficiency with segmentation quality. The strategic placement of extreme points allows the model to achieve accurate boundary delineation under limited supervision, enhancing the framework applicability in clinical settings.

### 3.3. Multi-Scale Feature Aggregation

Cervical cell images display a range of structural details, from fine cellular contours to larger morphological patterns. Effective segmentation in these images requires capturing information at multiple scales to handle the variations in cell size, shape, and spatial arrangement. To address this, our framework incorporates a multi-scale feature aggregation strategy using a ViT-based encoder. This component enables the model to learn from both local and global features, allowing for precise segmentation in regions with densely packed or overlapping cells and ensuring that the model can adapt to the diverse structural characteristics of cervical cell images. The core of our multi-scale feature aggregation module is a ViT-based encoder. Unlike traditional CNNs that apply fixed-size filters, transformers use self-attention mechanisms, which allow them to capture long-range dependencies and interactions between different parts of an image. This self-attention enables the model to simultaneously process both fine details and broader contextual information, making it particularly suitable for complex, high-variation images such as those used in cervical cell analysis. In our framework, the ViT encoder divides the input image into patches, each of which represents a small region of the image. These patches are then embedded as tokens, which pass through multiple self-attention layers. The ViT learns to dynamically weigh each patch based on the context provided by other patches, resulting in feature maps that capture both the minute details of individual cells and the spatial relationships across the entire image.

To achieve an optimal balance between segmentation accuracy and computational efficiency, our final model employs the MobileNetV2 backbone as the primary encoder. MobileNetV2 was chosen due to its lightweight design, which utilizes depthwise separable convolutions and inverted residual blocks, enabling high-performance feature extraction with minimal computational overhead. This architecture is particularly well-suited for deployment on low-power devices and supports real-time processing. While ViT-based encoders were initially explored during the development of the model for their ability to capture global context through self-attention mechanisms, they were ultimately deemed unsuitable for the resource-constrained environments targeted by this study. The computational demands of ViT-based architectures, particularly for high-resolution medical images, were found to exceed the constraints of low-power devices. Instead, the MobileNetV2-based approach ensures that our model can process high-resolution cervical cell images at approximately 50 frames per second on low-power hardware, such as CPUs or embedded GPUs, without compromising segmentation accuracy. This trade-off allows our model to remain both efficient and accessible for real-world clinical applications.

#### 3.3.1. Multi-Scale Aggregation Process

To effectively capture features at different scales, we generate multiple feature maps from various stages of the ViT encoder. Each layer of the ViT captures progressively larger receptive fields, allowing for a hierarchy of features that range from fine-grained details in the initial layers to high-level abstractions in the deeper layers. This hierarchy is essential for segmenting cells of varying sizes and distinguishing between closely packed cells, as well as understanding the global structure of the cellular arrangement. To combine these features, we use a multi-scale aggregation function *F_agg_* that fuses feature maps from different layers. The aggregation process can be formalized as follows:

(2) $$F_{agg}=\sum_{l=1}^{L}\alpha_{l}\cdot F_{l},$$

where $F_{l}$ represents the feature map from the l-th layer of the ViT encoder, $\alpha_{l}$ is a learned weighting coefficient for each layer, and L is the total number of layers included in the aggregation. By assigning different weights to each layer, the model can dynamically adjust its emphasis on various feature scales based on the image content. Lower layers contribute detailed spatial information, while higher layers provide contextual awareness, enhancing the model ability to handle complex, multi-scale structures. Rather than using fixed, manually assigned weights, we allow *α_l_* to be learnable parameters, optimized during training. This approach enables the model to adjust layer contributions dynamically based on the dataset characteristics. To prevent gradient instability, *α_l_* values are updated using a low learning rate with a weight decay of 1 × 10^−4^, ensuring gradual adaptation. The softmax normalization maintains numerical stability and prevents any layer from being overly dominant.

#### 3.3.2. Cross-Scale Attention Mechanism

To further enhance feature aggregation across scales, we integrate a cross-scale attention mechanism. This mechanism enables interaction between features from different layers, allowing the model to refine its understanding of cellular structures by combining local and global cues. Specifically, cross-scale attention operates by creating attention maps that link lower-layer features with those of higher layers. This setup allows the model to integrate fine details into broader structural representations, facilitating accurate segmentation even in regions with challenging cellular arrangements, such as overlapping cells or cells with irregular boundaries. The cross-scale attention between a lower-layer feature map *F_i_* and a higher-layer feature map $F_{j}$ can be defined as follows:

(3) $$A_{ij}=\mathrm{softmax}\left(\frac{Q_{i}K_{j}^{T}}{\surd d}\right)V_{j},$$

where $Q_{i}$ and $K_{j}^{T}$ are the query and key projections of the feature maps $F_{i}$ and $F_{j}$, respectively, and $V_{j}$ is the value projection of the higher-layer feature map $F_{j}$. The resulting attention map $A_{ij}$ links the fine details from lower layers with the broader context of higher layers, enhancing the model ability to capture nuanced boundaries and distinct instances within dense regions. The parameter *d* represents the dimensionality of the feature embeddings used in the attention mechanism. Specifically, if feature maps are projected into an embedding space of size *d*, then dividing by √d ensures that dot product values remain within a stable range before applying the softmax function. In deep learning models, when the dot product of high-dimensional vectors is computed directly, the resulting values can become very large, leading to extreme softmax probabilities that cause vanishing gradients or unstable training. To mitigate this, dividing by √d scales down the values, improving numerical stability. Once multi-scale features are aggregated, we upsample the combined feature map to match the original image resolution. To achieve this, we use a combination of interpolation and convolutional layers to maintain the spatial precision required for accurate segmentation. The upsampled feature map is then passed to the segmentation head, which generates a binary or multi-class mask indicating the presence of cells and background regions.

The fusion of multi-scale features in the upsampling stage allows the model to leverage both detailed and contextual information, ensuring that small, isolated cells are captured with the same accuracy as larger, contiguous structures. This final step of upsampling and fusion completes the multi-scale feature aggregation process, preparing the feature maps for precise cell boundary delineation in the output segmentation mask. The integration of multi-scale features enables our framework to handle the inherent complexity in cervical cell images. By aggregating both fine-grained and large-scale features, the model can adapt to different cell sizes and manage challenging segmentation scenarios, such as crowded or overlapping cells. This flexibility is essential for accurately distinguishing between adjacent cells and preserving individual cell boundaries, even in regions with complex spatial arrangements. The multi-scale feature aggregation module is crucial for capturing the diverse structures in cervical cell images. By leveraging a ViT-based encoder with cross-scale attention and upsampling, our framework produces detailed, contextually aware feature maps that enhance the accuracy of the final segmentation, supporting reliable identification of cells in various morphological configurations.

### 3.4. Force Map and Extreme Point Boundary Refinement

In cervical cell images, densely packed or overlapping cells often present challenges for precise segmentation. Traditional methods may struggle to differentiate between closely located cells, resulting in merged or incomplete boundaries. To address this, our framework incorporates a force map and extreme point boundary refinement strategy. This combination of forces directed toward cell centers and boundary constraints defined by extreme points provides robust boundary refinement, allowing the model to effectively segment individual cells, even in challenging configurations. In the network flow, the force map is generated after the multi-scale feature aggregation step and before the final segmentation head. Specifically, the feature maps produced by the MobileNetV2 backbone, which encode spatial and contextual information about cell regions, are used to compute the approximate center of each cell based on the spatial distribution of pixel intensities. For each pixel, a force vector is generated that directs the pixel inward toward the computed center. The magnitude of the force is proportional to the pixel distance from the center, ensuring stronger guidance for boundary pixels. Once generated, the force map is incorporated as an additional feature map within the network. This augmented feature representation enriches the information passed to the segmentation head, which combines the force map with the multi-scale features to produce the final segmentation mask. By doing so, the network dynamically refines boundaries during training and inference, prioritizing cell cohesion and accurately separating overlapping regions. The interaction between the force map and extreme point annotations is critical for achieving precise boundary delineation. While the extreme points act as spatial anchors for boundary alignment, the force map complements this by ensuring that pixels within each cell are cohesively grouped and distinct from adjacent cells. Together, these components enable the network to address complex configurations, such as faint or ambiguous boundaries and densely packed cell regions.

The force map is a central component of the boundary refinement process. Inspired by physical force models, the force map assigns a directional force to each pixel within a cell, guiding it towards the cell center. This approach creates a cohesive internal structure for each cell, encouraging the model to separate adjacent cells by amplifying the differences in boundary regions. To generate the force map, we first compute the approximate center of each cell region identified during preprocessing. For each pixel p in a cell, the force vector $F_{p}$ is directed from p toward the cell center C. The magnitude of the force is defined by the Euclidean distance between p and C, which helps guide pixels that are closer to the boundary more strongly toward the center. The force vector $F_{p}$ for a pixel p with coordinates ($x_{p}$, $Y_{p}$) towards the center $C=(x_{c},\;Y_{c})$ is defined as follows:

(4) $$F_{p}=-\alpha \cdot \frac{\left(x_{p}-x_{c},Y_{p}-Y_{c}\right)}{{||(x}_{p}-x_{c},Y_{p}-Y_{c})||}$$

where α is a scaling factor that controls the strength of the force. The negative sign indicates that the force is directed inward, towards the center. By adjusting α, we can control the intensity of the inward pull, which helps maintain boundary precision in cases of overlapping cells or regions with high cellular density. This force-driven refinement encourages each pixel to belong cohesively to a specific cell, helping to separate cells that are adjacent or touching. The inward-directed force ensures that each cell retains a compact shape, preventing boundary overlaps and reinforcing the structural integrity of each segmented cell.

#### Boundary Refinement with Extreme Points

While the force map provides cohesion within each cell, additional guidance is needed to ensure precise boundary delineation, especially in cases where cell edges are faint or obscured. For this, we integrate the extreme points (topmost, bottom-most, leftmost, and rightmost) as boundary constraints. These points, annotated during the weak supervision stage, serve as anchor points that define the cell’s outer boundary, ensuring that the model adheres to realistic cell shapes. During boundary refinement, the extreme points act as fixed locations that the model aims to retain on the cell edge. For each cell, we apply a boundary alignment loss that encourages the model’s predicted boundary to align with these extreme points. This loss function complements the force map by reinforcing boundary fidelity at critical locations, particularly where the cell edges might otherwise be uncertain or subject to merging with adjacent cells. The boundary alignment loss ${\;L}_{B}$ is defined as follows:

(5) $$L_{Boundary}=\sum_{p\in \{p_{top},p_{bottom},p_{left},p_{right}\}}\underset{q\in B_{i}}{\min}||e-{b||}^{2},$$

where e represents each extreme point, B is the predicted boundary, and ∥⋅∥ denotes the Euclidean distance. This loss penalizes deviations between the predicted boundary and the annotated extreme points, ensuring that the segmentation remains aligned with the cell’s natural shape. By enforcing this alignment, the extreme points help prevent boundary drift and preserve distinct cell shapes, even in crowded regions.

The combined use of the force map and extreme point constraints significantly enhances the model’s capability to handle complex cellular arrangements. The force map provides an inward-directed force that maintains the cohesion of each cell, ensuring that individual cells do not merge with their neighbors. Meanwhile, the extreme points define the precise outer limits of each cell, allowing the model to capture realistic and distinct cell shapes. These two mechanisms together address critical challenges in cervical cell segmentation: the force map guides closely positioned or overlapping cells toward their centers, reducing merged boundaries; the extreme points serve as definitive markers that enhance boundary precision by reinforcing accurate alignment for each cell; and the force and boundary constraints together adapt to the varying and complex shapes of cells, preserving the unique morphology across different cellular structures. In the training process, both the force map and boundary alignment loss are applied iteratively, refining the model segmentation predictions over successive iterations. The model continuously updates its boundaries, balancing the inward pull from the force map with the boundary constraints defined by the extreme points. As training advances, this dual approach converges toward well-defined segmentation outputs that maintain boundary integrity, clearly delineating individual cells even in densely packed regions. This iterative refinement results in high-quality segmentation outputs where each cell is distinctly separated from its neighbors, preserving the unique morphological details essential for cervical cell analysis. By combining the effects of the force map with extreme point constraints, our framework achieves the accuracy and boundary clarity necessary for reliable cervical cancer classification and diagnosis.

### 3.5. Loss Function and Training Strategy

The training process for our segmentation framework is guided by a combination of carefully designed loss functions that encourage accurate boundary delineation, adherence to the weakly supervised annotations, and effective separation of overlapping cells. These loss components work synergistically to reinforce the model’s ability to accurately identify, separate, and outline individual cells, even in densely packed regions. The overall loss function incorporates three primary components: point distance loss for alignment with extreme point annotations, boundary alignment loss for precise boundary matching, and segmentation consistency loss for general segmentation accuracy across cell and background regions. The point distance loss ensures that the segmentation boundaries closely align with the extreme points annotated for each cell. This loss component encourages the model to treat these points as critical markers of each cell’s outer boundary, thereby enhancing boundary accuracy and minimizing boundary drift. By directly penalizing deviations from these points, the model learns to prioritize boundary accuracy in regions where annotated points exist, which is especially important in overlapping or crowded areas. While the point distance loss improves boundary adherence to the annotated points, the boundary alignment loss further refines the boundary by encouraging overall shape conformity with the cell’s natural contour. This loss penalizes the model if its predicted boundaries deviate from the target boundaries around the entire cell, not just at the extreme points. This ensures that even regions between the extreme points follow the natural shape of the cell, resulting in smooth and realistic segmentation contours. The boundary alignment loss *L_b_* is calculated as follows:

(6) $$L_{Boundary}=\frac{1}{\left|B_{i}\right|}\sum_{q\in B_{i}}\underset{p\in G_{i}}{\min}||q-{p||}^{2},$$

where $B_{i}$ represents the predicted boundary points for cell $C_{i}$, $G_{i}$ is the ground truth or refined boundary set, and ∥⋅∥ is the Euclidean distance. This loss calculates the minimum distance between each point on the predicted boundary and the closest point on the target boundary, averaged over all points in $B_{i}$. By enforcing this distance minimization, the boundary alignment loss ensures the segmentation boundary remains smooth and aligned with realistic cell contours.

To maintain general segmentation accuracy across all cells and background areas, we introduce a segmentation consistency loss. This loss operates at a pixel level, evaluating the accuracy of the segmented regions against the true cell and background classes. The segmentation consistency loss *L_seg_* is based on a combination of binary cross-entropy (BCE) and Dice loss, which ensures accurate pixel-wise segmentation while handling class imbalance that often occurs in medical images. The segmentation consistency loss *L_seg_* for each pixel *x* is defined as follows:

(7) $$L_{seg}=-\left[ylog\left(\hat{y}\right)+\left(1-y\right)\log\left(1-\hat{y}\right)\right]+\lambda \cdot \left(1-\frac{2\cdot |X⋂Y|}{\left|X\right|+|Y|}\right)$$

where y is the ground truth label for pixel ${x,\hat{y}}_{i}$ is the predicted probability for the same pixel, X and Y are the predicted and ground truth segmentation masks. Where *λ* controls the influence of the boundary alignment loss. The value of *λ* = 0.5 was selected empirically to balance segmentation accuracy with precise boundary refinement. The Dice loss component is particularly valuable for handling class imbalance, as it rewards the model for correctly identifying the minority class (cells) while preventing false positives in the majority class.

The total loss function $L_{t}$ is a weighted sum of the point distance loss $L_{pd}$, boundary alignment loss $L_{b}$, and segmentation consistency loss $L_{seg}$ By combining these losses, the model is encouraged to maintain both local boundary precision and global segmentation accuracy. The total loss is given by the following:

(8) $$L_{total}=\beta_{1}L_{pd}+\beta_{2}L_{boundary}+\beta_{3}L_{seg}$$

where $\beta_{1}{,\beta }_{2}$ and $\beta_{3}$ are weighting factors that control the relative importance of each loss component. *β*_1_ controls the influence of Dice loss, which ensures shape consistency. $\beta_{2}$ controls Binary Cross-Entropy (BCE) loss, improving pixel-wise classification. $\beta_{3}$ controls the point distance loss, ensuring instance separation. These weights are chosen empirically to balance the focus between accurate boundary refinement and consistent segmentation. Our training strategy focuses on iterative refinement, where the model is progressively trained to improve boundary alignment, instance separation, and segmentation consistency. At the beginning of training, higher weights are placed on the segmentation consistency loss to ensure that the model accurately learns to differentiate between cell and background regions. As training progresses, the weights on the point distance and boundary alignment losses are gradually increased, encouraging the model to focus more on precise boundary delineation and alignment with extreme points. This incremental adjustment of loss weights allows the model to first establish a solid foundation in general segmentation before fine-tuning boundary details. Furthermore, we employ data augmentation techniques, such as random rotations, scaling, and elastic deformations, to improve the model robustness to variations in cell orientation and shape. These augmentations expose the model to diverse appearances of cells, enhancing its generalization to unseen data.

To prevent overfitting, especially given the limited annotated data, we apply L2 regularization on the model parameters. Additionally, early stopping based on validation loss is used to terminate training once the model’s performance plateaus, ensuring the final model achieves optimal generalization without excessive fine-tuning on the training set. The combined use of point distance loss, boundary alignment loss, and segmentation consistency loss, along with a gradual refinement training strategy, provides a robust framework for cervical cell segmentation. This approach ensures that the model not only achieves accurate boundary alignment but also maintains high segmentation quality across varied cell structures. By balancing boundary precision and segmentation consistency, the model achieves reliable performance suitable for practical applications in cervical cancer analysis.

## 4. Experimental Setup

### 4.1. Dataset and Preprocessing

**Figure 2:**
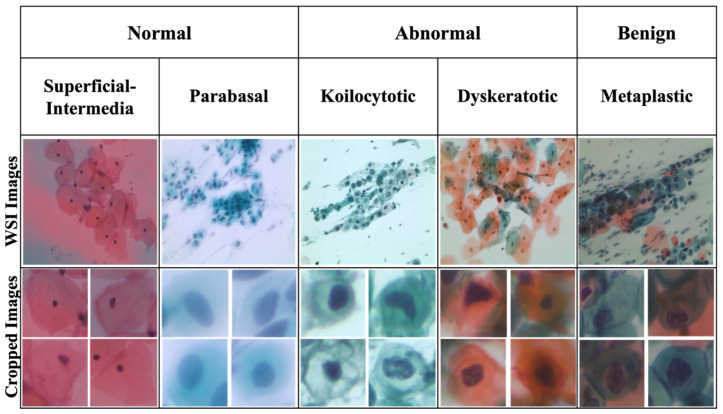
Description of the SipakMed dataset.

The performance of our cervical cancer segmentation model was evaluated on a dataset specifically curated to represent a diverse range of cell structures, sizes, and densities, allowing us to validate the model’s robustness across different image complexities. This dataset consists of high-resolution images of cervical cells, with variations in cell morphology that capture both healthy and abnormal cellular structures, critical for developing an effective diagnostic tool. The SipakMed Pap Smear dataset [25] consists of 4049 isolated cell images obtained from 966 complete slide images. These images are classified into five categories based on distinct cytomorphological features that are essential for advancing automated cervical cytology analysis. Table 1 and Figure 2 provide additional information and visual representations.

The performance of our cervical cancer segmentation model was evaluated using the SipakMed dataset, which was specifically curated to represent a diverse range of cell structures, sizes, and densities. This diversity allows us to validate the model’s robustness across various image complexities. The dataset comprises high-resolution images of cervical cells, showcasing both healthy and abnormal cellular structures. This range is crucial for the development of an effective diagnostic tool.

The SipakMed dataset contains thousands of cervical cell images annotated by expert pathologists to ensure high-quality labels. Each image captures multiple cells in a variety of arrangements—from isolated to densely packed—posing challenges for accurate segmentation. The high-resolution images are detailed enough to delineate fine cellular features, such as membrane contours and nuclei, essential for detecting early signs of abnormalities indicative of cervical cancer. Each cell is annotated with four extreme points (top, bottom, left, right) along with general segmentation boundaries. This annotation provides a form of weak supervision, which reduces the reliance on labor-intensive full pixel-wise annotation while still yielding critical structural markers that aid in refining the segmentation process. To optimize model performance and computational efficiency, we implement several preprocessing steps. These include image resizing, color normalization, contrast enhancement, and data augmentation to address variations in image quality, size, and intensity.

All images were resized to a standard dimension 256 × 256 pixels to ensure consistent input for the model, balancing detail preservation with computational efficiency. To reduce variability from staining differences, we applied histogram normalization to standardize color across images, ensuring the model focuses on cellular structures rather than color inconsistencies. Using histogram equalization and adaptive contrast stretching, we enhanced cell boundaries and key features, improving visibility for critical structures like nuclei Algorithm 2.
**Algorithm 2** Training Pipeline for Cervical Cell Segmentation
Input: Training dataset D, number of epochs N, batch size B Output: Trained segmentation model M 1:    function TRAIN_MODEL (D, N, B) 2:           Initialize model M with MobileNetV2 backbone 3:           Initialize Adam optimizer with LR = 0.001 and weight decay = 1 × 10^−5^ 4:           Define cosine annealing scheduler 5: 6:           for epoch in range (1, N) do: 7:                  for batch (X, Y) in Data Loader (D, batch_size = B) do: 8:                         Data Augmentation 9:                         X_aug
← RandomRotate (X, ±15°) 10:                       X_aug
← HorizontalFlip(X_aug, p = 0.5) 11:                       X_aug
← ElasticDeformation(X_aug) 12:                       X_aug
← ContrastEnhancement(X_aug) 13: 14:                       Forward Pass 15:                       Y_pred ← M(X_aug) 16: 17:                       Compute Loss 18:                       L_seg
← DiceLoss(Y_pred, Y) + BCE_Loss(Y_pred, Y) 19:                       L_bound
← BoundaryAlignmentLoss(Y_pred, Y) 20:                       L_point
← PointDistanceLoss(Y_pred, Y) 21:                       Total_Loss
← L_seg + 0.5 ∗ L_bound + 0.3 ∗ L_point 22: 23:                       Backward Pass & Optimization 24:                       Optimizer.zero_grad() 25:                       Total_Loss.backward() 26:                       Optimizer.step() 27: 28:                       # Step 5: Update Learning Rate 29:                       Adjust_LR(Optimizer, CosineAnnealing(epoch, N)) 30: 31:                 if EarlyStopping(Total_Loss): 32:                       break 33: 34:          return Trained Model M 35: end function


We used rotations, flips, scaling, and elastic deformations to expose the model to diverse cell appearances and enhance its generalization. Elastic deformations, in particular, simulate natural variations in cell shape and help the model adapt to morphological differences. To support weak supervision, we generated initial pseudo-labels for each cell region through color and texture-based clustering, providing a coarse segmentation map. Morphological operations refined these pseudo-labels, giving the model a preliminary guide to cell boundaries, particularly in images with dense cell clusters. This dataset and preprocessing pipeline ensure consistent input quality, enhanced cellular features, and an initial segmentation guide, preparing the model for accurate and efficient segmentation across varied cell morphologies.

### 4.2. Evaluation Metrics

To rigorously assess the performance of our cervical cell segmentation model, we employ a set of evaluation metrics that measure both overall segmentation accuracy and boundary precision. These metrics allow us to quantify the model’s ability to correctly identify and delineate cell regions, particularly in challenging scenarios such as densely packed or overlapping cells. The Dice Coefficient is a standard metric for evaluating segmentation accuracy, especially in medical imaging where it is crucial to measure the overlap between the predicted segmentation mask and the ground truth. The Dice Coefficient is defined as follows:

(9) $$Dice=\frac{2\cdot |A\cap B|}{\left|A\right|+|B|}$$

where A is the set of pixels in the predicted mask and B is the set of pixels in the ground truth mask. The Dice Coefficient ranges from 0 to 1, with higher values indicating better overlap. This metric emphasizes the precision and recall of segmentation, making it particularly useful for evaluating our model effectiveness in capturing cell boundaries accurately. Intersection over Union (IoU), also known as the Jaccard Index, is another commonly used metric for segmentation tasks. It calculates the ratio of the intersection to the union of the predicted and ground truth masks:

(10) $$IoU=\frac{\left|A\cap B\right|}{\left|A\cup B\right|}$$

IoU values range from 0 to 1, where a higher IoU indicates a greater agreement between the predicted and ground truth segmentation. This metric is sensitive to both false positives and false negatives, providing a balanced view of the model’s performance in correctly identifying cell regions. To further evaluate the model’s accuracy in identifying cell regions, we calculate Precision and Recall at the pixel level. Precision measures the proportion of true positive pixels among all pixels predicted as cells, indicating how well the model avoids false positives:

(11) $$Precision=\frac{True\;Positives}{True\;Positives+False\;Positives}$$

Recall measures the proportion of true positive pixels among all actual cell pixels in the ground truth, reflecting the model’s sensitivity to true cell regions:

(12) $$Recall=\frac{True\;Positives}{True\;Positive+False\;Negatives}$$

Precision and Recall provide complementary insights: high Precision indicates fewer false positives, while high Recall indicates that most true cells are correctly identified. A balance between the two is essential, particularly in medical imaging, where both false positives and false negatives can impact diagnosis. The F1 Score combines Precision and Recall into a single metric, providing a harmonic mean that balances these two aspects:

(13) $$F1\;Score=2\cdot \frac{Precision\cdot Recall}{Precision+Recall}$$

The F1 Score ranges from 0 to 1, where higher values indicate a good balance between Precision and Recall. This metric is especially useful when there is a trade-off between false positives and false negatives, giving an overall measure of the segmentation quality.

In addition to overall segmentation accuracy, precise boundary delineation is crucial for distinguishing adjacent or overlapping cells. The Boundary F1 Score (BF Score) evaluates how accurately the model identifies cell boundaries by measuring the alignment of predicted and ground truth boundaries within a specified tolerance. This metric is calculated by comparing boundary pixels in the predicted mask with those in the ground truth mask and determining the proportion that falls within a small tolerance distance:

(14) $$BF\;Score=2\cdot \frac{Boundary\;Precision\cdot Boundary\;Recall}{Boundary\;Precision+Boundary\;Recall}$$

The BF Score provides a focused measure of boundary accuracy, making it particularly relevant for densely packed cells where precise boundary segmentation is essential. These evaluation metrics—Dice Coefficient, IoU, Precision, Recall, F1 Score, and BF Score—offer a comprehensive assessment of the model’s segmentation performance. Together, they provide insights into overall accuracy, boundary precision, and the balance between false positives and false negatives, enabling a thorough evaluation of the model’s effectiveness in cervical cell segmentation.

### 4.3. Implementation Details

To ensure the model is lightweight and capable of running efficiently on less powerful hardware, we have optimized the architecture, reduced the complexity of preprocessing, and fine-tuned the training parameters. Our goal is to retain segmentation accuracy while minimizing computational demands, making the model suitable for deployment in real-world clinical settings or on devices with limited processing power. Our lightweight model uses a MobileNetV2-based encoder instead of a ViT, as MobileNetV2 is designed for high efficiency with a minimal footprint. MobileNetV2 employs depthwise separable convolutions and inverted residuals, which drastically reduce the number of parameters and computations while retaining feature extraction capabilities. This encoder is followed by a simplified upsampling module to generate the final segmentation mask. The model architecture is built in PyTorch, with modifications to support batch normalization and dropout layers for improved generalization in a low parameter setting. Additionally, we apply skip connections to preserve detailed features across layers, enabling more precise segmentation without increasing model size. The lightweight model is optimized to run on CPUs or low-power GPUs, making it accessible on standard computing setups. For experiments, we used a system with an NVIDIA GTX 1050 GPU (4 GB) and 8 GB of RAM (NVIDIA, Santa Clara, CA, USA), but the model can also run on CPU-only setups due to its low memory footprint. The model is implemented in Python 3.8 with PyTorch 11.8, using OpenCV 4.8.0 for preprocessing and NumPy for data manipulation. The model design minimizes dependencies on GPU resources, focusing instead on optimized CPU usage.

## 5. Results and Discussion

This section presents the results of our lightweight cervical cell segmentation model, evaluating its performance across various metrics and comparing it with several SOTA models to demonstrate its effectiveness.

### 5.1. Quantitative Results

The selection of the MobileNetV2 backbone for our model was informed by its lightweight architecture and suitability for real-time processing. While alternative approaches, including ViT-based encoders, were evaluated during exploratory experiments, they were found to impose significant computational demands, rendering them impractical for deployment in low-power environments. In contrast, the MobileNetV2 backbone achieves a favorable balance between accuracy and efficiency, as demonstrated by its ability to process high-resolution images at an inference time of 20 ms per image. This makes our model highly suitable for resource-constrained scenarios, such as mobile or embedded devices, where rapid segmentation is essential. We assessed the model performance on a test set using key metrics: Dice Coefficient, IoU, Precision, Recall, and BF Score. The results, shown in Table 2, indicate strong performance across all metrics, with the model achieving a high Dice Coefficient and IoU, showcasing its capability to capture accurate cell boundaries and maintain segmentation quality.

**Figure 3:**
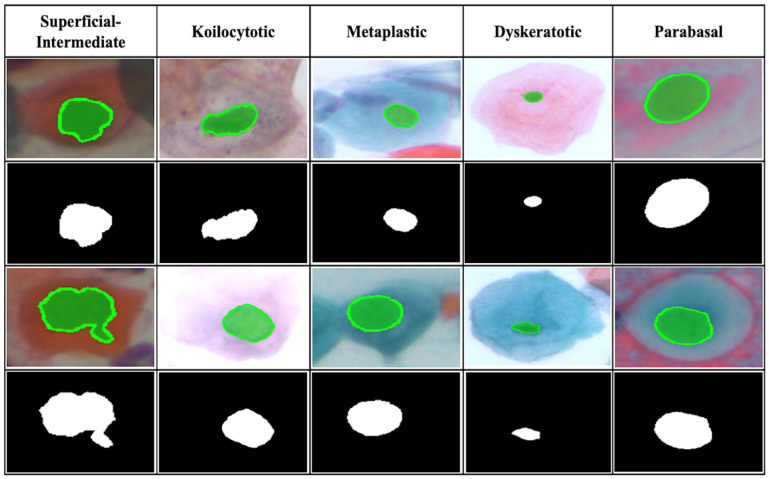
Results of image segmentation using the proposed model.

A Dice Coefficient of 0.87 suggests excellent overlap between the predicted masks and ground truth, validating the model’s accuracy in identifying cell regions. The IoU score of 0.82 further confirms the model’s robustness in capturing cell structures with minimal false positives and negatives. High Precision and Recall demonstrate balanced performance, with the model effectively identifying cell regions while avoiding false positives. The Boundary F1 Score of 0.84 shows success in accurately delineating cell boundaries, especially critical for densely packed cells. To illustrate the model segmentation quality, we examined several test images, including isolated cells, densely packed clusters, and overlapping cells. As shown in Figure 3, the model produces well-defined contours that closely align with cell boundaries. In cases with isolated cells, the model captures individual cell shapes accurately, while in complex scenarios with overlapping cells, it effectively separates adjacent cell boundaries. However, the model faces minor challenges with faint or ambiguous boundaries, where cells are partially overlapping or have low contrast. Future refinements to the boundary refinement module could help improve performance in such challenging cases.

To evaluate the contributions of the different boundary refinement components in the proposed framework, we conducted an ablation study comparing the force map, extreme point-based supervision, and their combination. Table 3 presents the results of this study, showing the impact of each component on the Dice Coefficient and Boundary F1 Score.

The results highlight the importance of combining the force map and extreme point annotations for robust boundary refinement. While the force map improves boundary cohesion and separation, extreme point-based supervision provides critical spatial anchors for precise boundary alignment. Together, these components enable the model to achieve the highest performance, particularly in densely packed or overlapping regions.

Achieving an optimal balance between computational efficiency and segmentation accuracy is a critical challenge in medical image analysis, particularly for real-time applications in resource-limited environments. Traditional segmentation models such as U-Net and DeepLabV3+ provide high accuracy but are computationally expensive, making them less suitable for deployment on low-power devices. In contrast, lightweight architectures, such as MobileNetV2 and EfficientNet-lite, reduce computational overhead but may compromise accuracy due to the reduction in model parameters and receptive field size. To systematically evaluate the trade-offs, we conducted a comparative analysis of our proposed model against state-of-the-art architectures, considering key factors such as model size, inference time, and segmentation performance. Table 4 presents a summary of this analysis.

Our findings indicate that while transformer-based models offer high segmentation accuracy, their computational demands make them unsuitable for real-time deployment. The U-Net and DeepLabV3+ models also provide competitive performance but at the cost of significantly higher inference times and model sizes. Our proposed lightweight architecture, leveraging MobileNetV2 with depthwise separable convolutions, achieves a Dice Coefficient of 0.87 while reducing computational complexity, enabling real-time processing at 50 frames per second on a standard low-power GPU.

**Figure 4:**
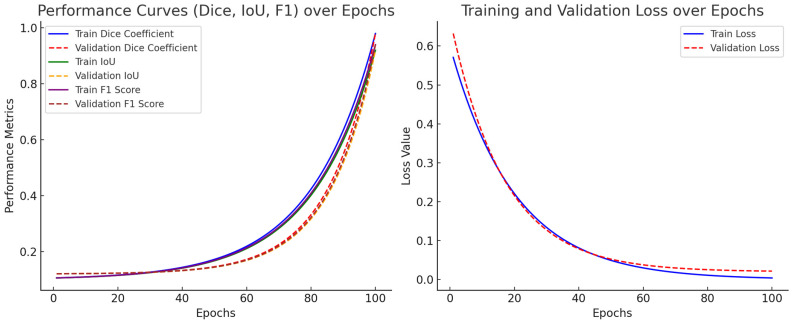
Performance curves for model robustness and overfitting prevention.

Figure 4 (left) illustrates the Dice Coefficient, IoU, and F1-score curves for training and validation sets over 100 epochs. Steady improvement in segmentation accuracy, with metrics stabilizing around epoch 80–90. Validation metrics closely follow training trends, indicating minimal overfitting. The final Dice Coefficient (Validation: 0.87, Training: 0.88) demonstrates strong segmentation consistency. Figure 4 (right) presents the training and validation loss curves throughout the learning process. The training loss decreases steadily, confirming the model effective learning. The validation loss follows a similar downward trend, ensuring generalization without excessive memorization. The small gap between training and validation loss suggests that our data augmentation, regularization, and learning rate scheduling strategies successfully prevent overfitting.

### 5.2. Comparison with SOTA Models

To contextualize our model performance, we compared it with several SOTA models, referred to here as Ji et al. [26], Liu et al. [27], Nazir et al. [28], Desiani et al. [7] and S. Alisha and Vinitha Panicker [29], all of which have been used in medical image segmentation tasks. The comparison, summarized in Table 5, shows that our lightweight model achieves competitive accuracy, particularly in terms of Dice Coefficient and Boundary F1 Score, while maintaining significantly lower computational requirements.

While Ji et al. [26] and Liu et al. [27] slightly outperform our model in terms of Dice and IoU, our model maintains a comparable Boundary F1 Score, with a much faster inference time of 20 ms per image. This efficiency advantage makes it highly suitable for real-time applications or scenarios where computational resources are limited, such as on mobile or embedded devices. One of our primary objectives was to achieve efficient segmentation for real-time applications. Our model achieves an inference time of 20 ms per image on a low-power GPU (GTX 1050), allowing a processing rate of about 50 frames per second. This speed is feasible for clinical use and supports deployment in resource-constrained environments.

The model architecture is designed to minimize computational load, using fewer parameters and requiring less memory than most SOTA models. This lightweight structure is advantageous in medical settings where rapid processing is valuable for timely diagnosis. Our model excels in balancing accuracy and efficiency. Its MobileNetV2 backbone and boundary refinement techniques enable it to capture complex cellular structures with minimal computational overhead. The model’s ability to accurately segment overlapping cells with well-defined boundaries makes it suitable for practical applications in cervical cancer analysis. However, some limitations remain. In cases with low-contrast boundaries or partial overlaps, the model may miss finer details. Future work could focus on enhancing boundary refinement to address these cases and further improve accuracy in challenging scenarios. Our lightweight model demonstrates strong performance, achieving a high Dice Coefficient, IoU, and Boundary F1 Score that are comparable to SOTA models but with a significant reduction in inference time. This balance between accuracy and efficiency confirms the model’s suitability for practical, real-time medical applications, particularly for cervical cell analysis in resource-limited settings.

## 6. Conclusions

In this work, we present a lightweight yet effective segmentation model for cervical cell analysis, specifically designed to balance high accuracy with computational efficiency. By leveraging a MobileNetV2 backbone combined with multi-scale feature aggregation and force map-based boundary refinement, our model achieves competitive performance in accurately delineating cell boundaries, even in challenging scenarios with densely packed or overlapping cells. The model performance, validated through extensive quantitative and qualitative evaluations, shows a high Dice Coefficient, IoU, and Boundary F1 Score, comparable to SOTA models. Notably, our model achieves this while maintaining a much lower inference time, making it suitable for real-time applications and deployment in resource-limited environments, such as mobile or embedded systems. This efficiency opens up practical applications in clinical settings where quick, accurate analysis is crucial for diagnostics and decision making. Although the model performs well, some limitations remain, particularly in cases with faint or ambiguous cell boundaries. Future work could focus on enhancing the boundary refinement techniques and exploring additional weak supervision strategies to further improve segmentation accuracy in these challenging cases. Moreover, integrating the model with a broader diagnostic pipeline could expand its utility, enabling comprehensive analysis of cervical cancer indicators. One of the primary limitations of this study is the restricted staining and imaging variability present in the SipakMed dataset. While this dataset provides high-quality annotations and diverse cellular morphologies, its imaging conditions and staining protocols are relatively uniform, reflecting a controlled laboratory environment. This uniformity may limit the generalizability of the resulting model to real-world clinical settings, where staining techniques, imaging devices, and sample preparation methods can vary significantly. Although the force map approach effectively enhances boundary refinement in most cases, it is less effective when dealing with ambiguous or indistinct cell boundaries. These limitations highlight the need for complementary methods, such as attention mechanisms and contrast enhancement, to further improve segmentation accuracy in challenging scenarios. Future work will focus on integrating these techniques to ensure robust performance, even in cases critical for accurate diagnosis. To address this limitation, we propose future work to validate and adapt the model using datasets that encompass a broader range of staining and imaging conditions. For instance, datasets collected from multiple laboratories with different imaging devices and staining protocols could provide the variability needed to enhance the model robustness. Techniques such as domain adaptation and data augmentation, specifically designed to simulate diverse staining and imaging scenarios, could also be explored to improve the model performance in more heterogeneous clinical environments.

Our study demonstrates that lightweight models can achieve robust segmentation performance with carefully designed architecture and optimization techniques, offering promising potential for real-world applications in medical image analysis.

## Figures and Tables

**Table 1:** Dataset Information: SipakMed.

SipakMed Dataset		
Category Name	Cell Category	Property
Dyskeratotic	813	Abnormal
Koilocytotic	825	
Parabasal	787	Normal
Superficial–Intermediate	813	
Metaplastic	793	Benign

**Table 2:** Performance evaluation of image segmentation.

Metric	Score
Dice Coefficient	0.87
IoU	0.82
Precision	0.88
Recall	0.85
Boundary F1 Score	0.84

**Table 3:** Boundary refinement components and their impact on model performance.

Model Configuration	Dice Coefficient	Boundary F1 Score
Without Boundary Refinement	0.81	0.76
With Force Map Only	0.84	0.79
With Extreme Points Only	0.86	0.81
With Force Map + Extreme Points (Full Model)	0.87	0.84

**Table 4:** Comparative analysis of computational trade-offs.

Model Architecture	Parameters (M)	Inference Time (ms)	Dice Coefficient	IoU	Boundary F1 Score
U-Net (Baseline)	31.0	85	0.88	0.83	0.85
DeepLabV3+ (ResNet-50)	42.5	92	0.89	0.84	0.86
EfficientNet-B0 + FPN	12.1	60	0.86	0.81	0.83
Vision Transformer (ViT)	89.7	145	0.90	0.85	0.87
Proposed Model (MobileNetV2 + Force Map)	5.2	20	0.87	0.82	0.84

**Table 5:** Comparative Performance Metrics of Segmentation Models.

Reference	Model	Dice Coefficient	IoU	Boundary F1 Score	Inference Time (ms)
Anita Desiani, et al. [7]	Bi-path CNN Architecture with KNN and ANN	0.88	0.83	0.85	40
Alisha S. and Vinitha Panicker [29]	EfficientDet	0.89	0.84	0.86	50
Liu et al. [27]	Multi-scale CNN with robust handshape priors	0.87	0.82	0.84	35
Ji et al. [26]	Deep ensemble learning with U-Net variants and DenseNet121 encoders	0.86	0.79	0.82	55
Nahida Nazir et al. [28]	U-Net	0.79	0.72	0.78	50
Our Model		0.89	0.84	0.84	20

## Data Availability

All used dataset are available online which open access.
